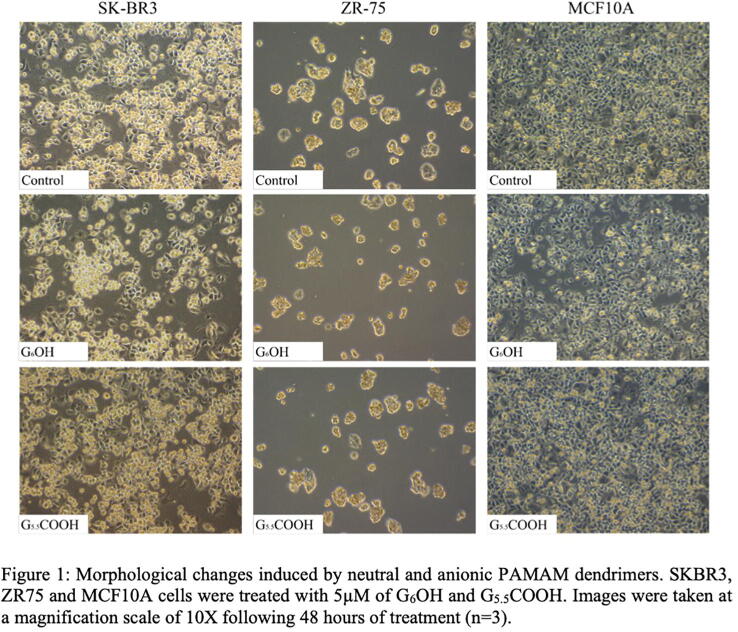# Substantial cell apoptosis provoked by naked PAMAM dendrimers in HER2-positive human breast cancer via JNK and ERK1/ERK2 signalling pathways

**DOI:** 10.1016/j.csbj.2021.05.011

**Published:** 2021-05-07

**Authors:** Hadeel Kheraldine, Ishita Gupta, Hashim Alhussain, Aayesha Jabeen, Farhan S. Cyprian, Saghir Akhtar, Ala-Eddin Al Moustafa, Ousama Rachid

**Affiliations:** a College of Pharmacy, QU Health, Qatar University, PO Box 2713, Doha, Qatar; b College of Medicine, QU Health, Qatar University, PO Box 2713, Doha, Qatar; c Biomedical Research Center, Qatar University, PO Box 2713, Doha, Qatar; d Biomedical and Pharmaceutical Research Unit, QU Health, Qatar University, PO Box 2713, Doha, Qatar; e Oncology Department, Faculty of Medicine, McGill University, Montreal, QC H3G 2M1, Canada

**Keywords:** 7-AAD, 7-amino-actinomycin D, Bax, Bcl-2 Associated X, Bcl-2, B cell lymphoma-2, EGFR, Epidermal growth factor receptor, ErbB2, erythroblastic oncogene B, ERK, Extracellular-signal-regulated kinase, FBS, Fetal bovine serum, FITC, Fluorescein isothiocyanate, GAPDH, Glyceraldehyde 3-phosphate dehydrogenase, JNK, c-Jun N-terminal kinase, PAMAMs, poly(amidoamine) dendrimers, PE, Phycoerythrin, PVDF, Polyvinylidene difluoride, PAMAMs, HER2-positive, Breast cancer, Chemoprevention, Apoptosis

## Abstract

HER2-positive breast cancer is one of its most challenging subtypes, forming around 15–25% of the total cases. It is characterized by aggressive behavior and treatment resistance. On the other hand, poly (amidoamine) (PAMAM) dendrimers are widely used in drug delivery systems and gene transfection as carriers. PAMAMs can modulate gene expression and interfere with transactivation of the human epidermal growth factor receptor family members (HER1-4). Nevertheless, the outcome of PAMAMs on HER2-positive breast cancer remains unknown. Thus, in this study, we investigated the anti-cancer effects of different generations of PAMAM dendrimers (G_4_ and G_6_) and the outcome of their surface chemistries (cationic, neutral, and anionic) on HER2-positive breast cancer cell lines, SKBR3 and ZR75. Our data showed that PAMAM dendrimers, mainly cationic types, significantly reduce cell viability in a dose-dependent manner. More significantly, PAMAMs induce substantial cell apoptosis, accompanied by the up-regulation of apoptotic markers (Bax, Caspases-3, 8 and 9) in addition to down-regulation of Bcl-2. Moreover, our data pointed out that cationic PAMAMs inhibit colony formation compared to controls and other types of PAMAMs. The molecular pathway analysis of PAMAM exposed cells revealed that PAMAMs enhance JNK1/2/3 expression while blocking ERK1/2, in addition to EGFR1 (HER1) and HER2 activities, which could be the major molecular pathway behind these events. These observed effects were comparable to lapatinib treatment, a clinically used inhibitor of HER1 and 2 receptors phosphorylation. Our findings implicate that PAMAMs may possess important therapeutic effects against HER2-positive breast cancer via JNK1/2/3, ERK1/2, and HER1/2 signalling pathways.

## Graphical abstract

**Figure:**
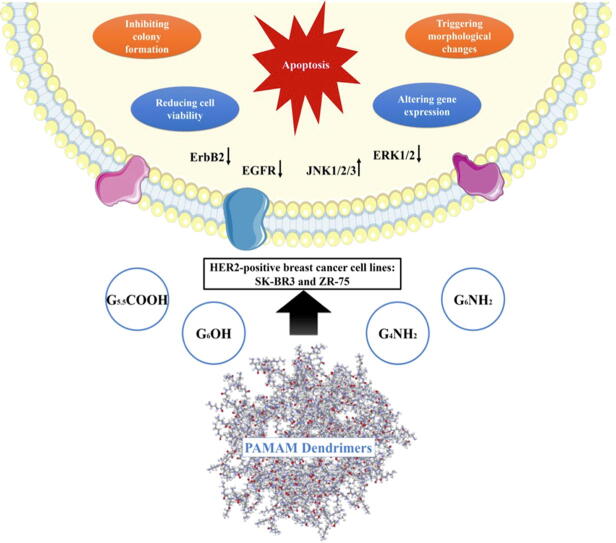

## 1 Introduction

Breast cancer is the most common type of cancer among women worldwide, with increased incidence and mortality rates [1]. Gene expression profiling classified breast cancer into four molecular subtypes; Luminal (A and B), HER2, basal-like and normal-like using hierarchical cluster analysis [2]. Of all subtypes, around 15–25% of total breast cancer cases are HER2-positive, where the human epidermal growth factor receptor type 2 (HER2) is overexpressed [3], [4]. Despite anti-HER2 and cytotoxic chemotherapy, HER2 subtype exhibits many challenges, including aggressive behavior, early relapse, poor prognosis, and higher recurrence rate [4]. Furthermore, along with hormonal therapy, current treatments for HER2-positive breast cancer include trastuzumab (a monoclonal antibody) and lapatinib (a tyrosine kinase inhibitor); however, they exhibit many limitations, mainly cardiac complications and chemo-resistance [5], [6], [7], [8], [9]. Thus, it is important to investigate new potential compounds as therapeutic agents for the treatment of HER2-positive breast cancer.

Advanced approaches in nanotechnology have enabled the production of compounds ranging from single nanoparticles to complex polymers with an extensive range of applications in drug delivery [10]. The role of polymers as active compounds for therapeutic applications has dominated the pharmaceutical polymers field. This area of research is called “polymer therapeutics”, where biologically active polymers can behave either as bioactive compounds or as inert carriers conjugated to a drug(s) [11]. One of the most functional nano-sized polymeric architectures is poly (amidoamine) dendrimers (PAMAMs), which are characterized by their surface chemistry and size (generation). PAMAM dendrimers are used in several biomedical applications, mainly in drug delivery systems and gene transfection [12], [13], [14]. Interestingly, it was found that naked PAMAM dendrimers themselves are not inert and may act as nano drugs for several conditions [15], [16], [17], [18]. One of their most important biological effects is their ability to modulate gene expression patterns and interfere with cell signaling pathways of epidermal growth factor receptor family; particularly EGFR and HER2 [16], [18], [19], [20]. These biological effects produced by PAMAM dendrimers raise the question of whether these polymers have a beneficial anti-HER2 effect in HER2-positive breast cancer.

Although several studies have explored the cytotoxic role of PAMAM-drug conjugate in cancers [21], [22], [23], [24], [25], the impact of naked PAMAM particles in breast cancer, especially HER2-positive subtype, remains unknown. In this study, we investigated the potential anti-cancer activities of different generations and surface chemistries of PAMAM dendrimers in HER2-positive breast cancer cells.

## 2 Materials and methods

### 2.1 Drugs and reagents

PAMAM dendrimers (G_4_NH_2_, G_6_NH_2_, G_6_OH, G_5.5_COOH) were synthesized by Dendritech and purchased from Sigma Aldrich Chemical Company (USA). Lapatinib (N-[3-chloro-4-[(3-fluorophenyl) methoxy] phenyl]-6- [5- [(2-methylsulfonyl ethyl amino) methyl]-2-furyl] quinazolin-4-amine) was purchased from LC Laboratories, Massachusetts, USA (L-4804).

### 2.2 Cell culture

HER2-positive breast cancer cell lines SKBR3 and ZR75 were purchased from the American Type Tissue Culture (ATCC) (Rockville, MD, USA). Cells were cultured and grown in complete cell culture media Gibco® RPMI-1640 (Gibco, Life Technologies) supplemented with 10% fetal bovine serum (FBS; Invitrogen, Life Technologies) and 1% PenStrep antibiotic (Invitrogen, Life Technologies). MCF10A cells were purchased from the American Type Tissue Culture (ATCC) (Rockville, MD, USA) and used as a control in this study. MCF10A cells were grown in 1X Gibco® DMEM, high glucose, GlutaMAX™ (1X) media (Gibco, Life Technologies) supplemented with 10% fetal bovine serum and 1% PenStrep antibiotic (Thermo Fisher Scientific, USA). Cells were maintained at 37 °C and 5% CO2 atmosphere. All the experiments were carried out when cells were ~ 70–80% confluent.

### 2.3 Cell viability assay

SKBR3, ZR75 and MCF10A cell lines (10,000 cells/well) were seeded on clear bottom 96-well plates (Thermo Fisher Scientific, USA), cultured in their respective media (100 µl/well) and were left to adhere overnight.

Cells were treated with different concentrations of PAMAM dendrimers, ranging from 0.1 to 100 µM. Additionally, lapatinib; a well-known anti-HER2 drug, was used as a control. Based on previous studies, lapatinib treatment was given in the concentrations of 10 to 100 nM in SKBR3, and 1 to 100 µM in ZR75 [27], [28], [29]. Cells were treated at three different time-points (24, 48, and 72 h). Control wells received 100 μl of media (control). Alamar Blue Cell viability reagent (Invitrogen, Thermo Fisher Scientific, USA) was used to determine cell viability, according to the manufacturer’s protocol. Briefly, 2% Alamar Blue dye was added to the plates, followed by incubation for 3–4 h. Post-incubation, fluorescence was recorded at a wavelength of 560 nm (excitement) and 600 nm (emission) using Infinite m200 PRO fluorescent microplate reader (TECAN, Switzerland).

### 2.4 Annexin V apoptosis assay

Apoptosis assay was performed using the PE Annexin V Apoptosis Detection Kit − 559,763 (BD Biosciences, USA) per manufacturer’s instructions. SKBR3 and ZR75 cells (1 × 10^6^ cells/dish) were seeded in 100 mm petri dishes (Thermo Fisher Scientific, USA) and left to adhere overnight. Cells were treated with PAMAM dendrimers as well as with lapatinib for 48 h. Cell populations were harvested, collected by trypsinization and washed with ice-cold PBS. Then, cells were resuspended in 200 µl of binding buffer. PE Annexin V apoptosis Detection Kit (BD Pharmingen, USA) was used to quantify cell apoptosis in treated versus untreated cells as per the manufacturer’s protocol. Briefly, 5 µl of PE Annexin V-and 5 µl of 7-AAD were added to the samples for 15 min in the dark. Controls were stained with PE Annexin V (no 7-AAD) and 7-AAD (no PE Annexin V). Samples were analyzed by Accuri C6 flow cytometer (BD Biosciences, USA). Data and figures were processed using the FlowJo V10 software and presented as density plots of PE Annexin V and 7-AAD staining.

### 2.5 Soft agar colony formation assay

The ability of cancer cells to form colonies in-vitro prior and post-treatment was assessed using soft agar growth assay. A total of 1.5 × 10^3^ cells of SKBR3 and ZR75 were placed in their medium containing 0.3% agar with/without PAMAMs (treated and control cells, respectively) and plated in a 6-well plate covered with a layer of 0.4% agar prepared in RPMI-1640 medium. Colony formation was monitored every two days for a period of three weeks. Then, colonies in each well were counted using the microscope.

### 2.6 RNA extraction and RT-PCR

RNA extraction kit (QIAGEN Canada Inc., ON, Canada) was used according to the manufacturer’s protocol to extract total RNA from SKBR3 and ZR75 cells treated with PAMAM dendrimers as well as with lapatinib. RNA concentrations were obtained using the nanodrop reader (ThermoFisher Scientific, USA) and samples were stored at −80° C for further analysis. RT-PCR was performed using The SuperScript® III One-Step RT-PCR System with Platinum® Taq DNA Polymerase (Invitrogen, USA). Samples were incubated in the Proflex Thermal Cycler (Thermo Fisher Scientific, USA) for reverse transcription at 60 °C for 30 min, initial PCR activation step 94 °C for 2 min followed by 40 polymerase chain reaction cycles. Each cycle consisted of 94˚C for 15 s, annealing temperature for 30 s, and 68˚C for 1 min. Final annealing was at 68˚C for 5 min. The oligonucleotide-specific primers for our genes of interest were used in this study (Table 1).

**Table 1:** Sequence of the oligonucleotide specific primer sets used for RT-PCR.

Gene	Forward Primer (5′-3′)	Reverse Primer (5′-3′)	Annealing Temperature (Ta) °C
Bax	GCTGCAGACATGCTGTGGATC	TCACAGCCAGGAGAATCGCAC	56
Bcl-2	GGATGCCTTTGTGGAATTGT	GTCCAAGATAAGCGCCAAGA	42
Caspase-3	GCAGCAAACCTCAGGGAAAC	TGTCGGCATACTGTTTCAGCA	50
Caspase-8	TCCTCTTGGGCATGACTACC	TGTCAATCTTGCTGCTCACC	56
Caspase-9	AGCCAGATGCTGTCCCATAC	CAGGAGACAAAACCTGGGAA	50
GAPDH	CCTCTCTGGCAAAGTCCAAG	CATCTGCCCATTTGATGTTG	56

The PCR product from each exon was visualized using 1.5% agarose gel (Promega, USA) run at 110 V, 400 mA for 40 min. Gels were imaged using the iBright CL1000 imaging system. To explore the gene expression associated with cell apoptosis, relative quantification was obtained by analyzing acquired images using ImageJ software (National Institutes of Health, Bethesda, MD, USA). The intensity of the bands relative to GAPDH bands was used to calculate the relative gene expression in each cell line. Fold change in the gene expression was calculated as the ratio between the value of treated samples over that of control ones.

### 2.7 Western blotting

Expression levels of proteins involved in apoptosis were analyzed by Western blotting as previously described by our group.[26] In brief, SKBR3 and ZR75 cells were seeded in 100 mm petri dishes (Thermo Fisher Scientific, USA) and left to adhere overnight. Cells were treated with PAMAM dendrimers as well as lapatinib for 48 h. Cell lysates were collected and quantified using Pierce BCA Protein Assay Kit (Thermo Fisher Scientific, USA) according to the manufacturer’s protocol. NuPAGE® Bis-Tris Electrophoresis System (Thermo Fisher Scientific, USA) was used to run Western blotting. Briefly, stained protein samples were boiled at 95 °C for 10 min and equal amounts of protein (50 μg) were resolved in NuPAGE® Novex® Bis-Tris Gels (4–12%) (Thermo Fisher Scientific, USA) and electroblotted onto PVDF membranes, followed by blocking with 3% BSA (Thermo Fisher Scientific, USA). Then PVDF membranes were probed with the following primary antibodies; mouse anti-Bcl-2 (Abcam: abID# ab692), rabbit anti-Caspase-3 (Abcam: abID# ab13847), mouse anti-ErbB2 (Abcam: abID# ab16901), rabbit anti-phosphorylated ErbB2 for endogenous levels of ErbB2 at Tyrosine 877 (Abcam: abID# ab47262), rabbit anti-EGFR (Abcam: abID# ab131498), rabbit anti-phosphorylated EGFR on Tyrosine 1068 (Abcam: abID# ab40815), rabbit anti-ERK1/ERK2 antibody (Abcam: abID# ab115799), rabbit anti ERK1 (phospho Tyrosine 202) + ERK2 (phospho Tyrosine 185) (Abcam: abID# ab201015) and rabbit anti-JNK1/JNK2/JNK3 (Abcam: abID# ab179461). To ensure equal loading of protein samples, the membranes were re-probed with rabbit anti-GAPDH (Abcam: abID# ab9485). Following primary antibody staining, membranes were incubated with an anti-rabbit IgG-HRP (cat. no: 7074S, Cell Signaling Technology, Inc.) or anti-mouse IgG-HRP (cat. no: 7076S, Cell Signaling Technology, Inc.) secondary antibody. Immunoreactivity was detected using Pierce™ ECL Western Blotting Substrate (Peirce Biotechnology) by chemiluminescence. Blots were imaged using the iBright^TM^ CL1000 imaging system (Thermo Fisher Scientific, USA).

Relative quantification of protein expression was obtained by analyzing acquired Western blotting images using ImageJ software (National Institutes of Health, Bethesda, MD, USA). The intensity of the bands relative to GAPDH bands was used to calculate a relative expression of proteins in each cell line.

### 2.8 Statistical analysis

Data were shown as an average of mean ± SEM (standard error of the mean). Each experiment was repeated at least three times (n = 3). One-way ANOVA followed by Tukey’s post-hoc test was used to compare the difference between treated and untreated cells. The data were analyzed using Microsoft Excel and GraphPad Prism software and differences with *p* < 0.05 were considered significant.

## 3 Results

**Fig. 1:**
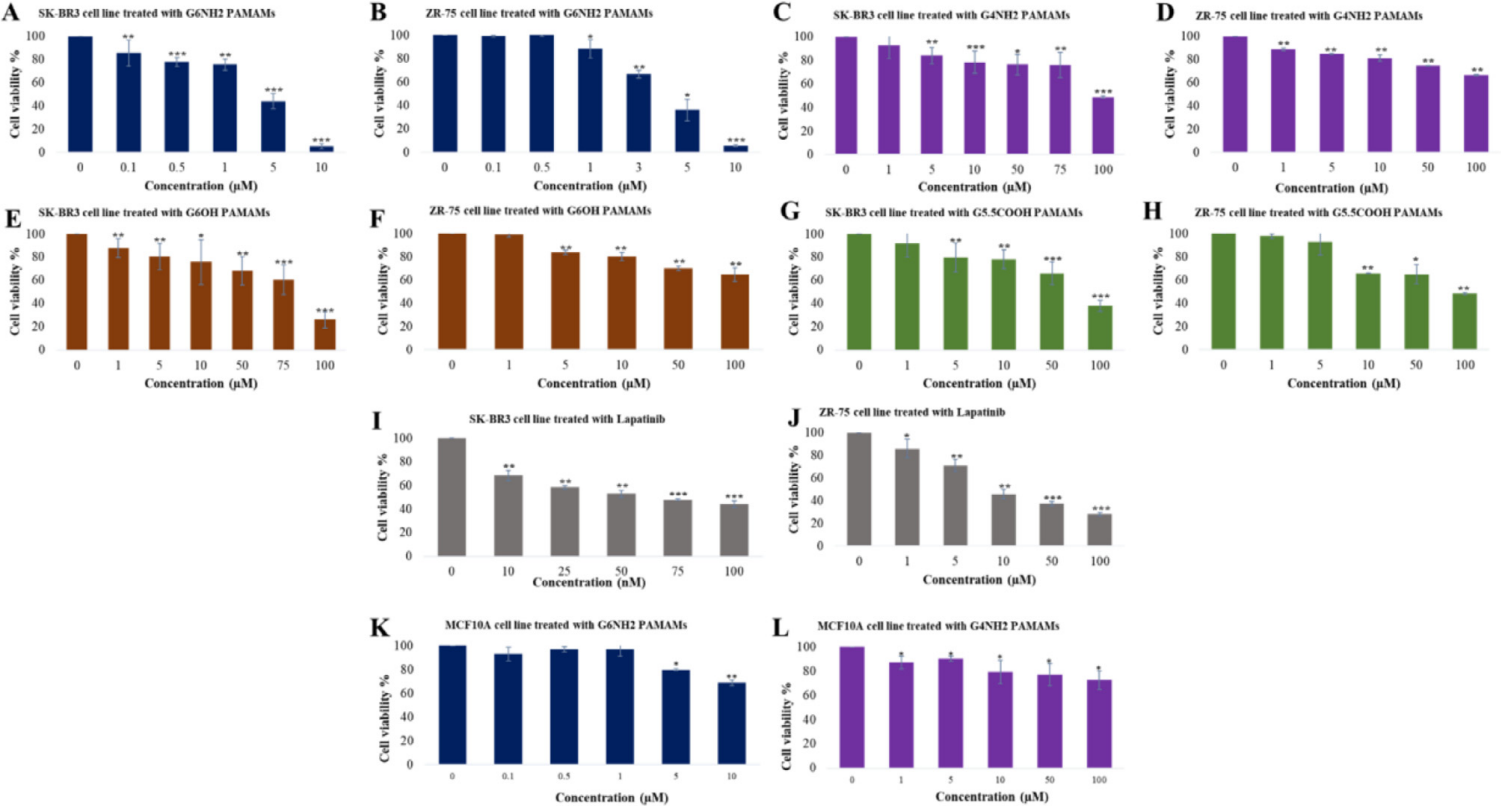
**(A-L).** Effect of different concentrations of **(A and B)** G_6_NH_2_ PAMAMs, **(C and D)** G_4_NH_2_ PAMAMs, **(E and F)** G_6_OH PAMAMs, **(G and H)** G_5.5_COOH PAMAMs, and **(I and J)** lapatinib on cell viability of SKBR3 and ZR75 cells after 48 h of treatment. Effect of G_6_NH_2_ and G_4_NH_2_ PAMAMs on cell viability of MCF10A cells **(I and J)** after 48 h of treatment. Data are presented as a percentage of treatment relative to the control (Mean ± SEM; n = 3). Statistical analysis was performed using one-way analysis of variance (ANOVA). Tukey’s post-hoc test was conducted to compare treatment groups and results were stated as *statistically significant when *p* < 0.05 compared to the control. * p < 0.05, **p < 0.01, and *** p < 0.001.

The effect of PAMAM dendrimers (G_4_NH_2_, G_6_NH_2_, G_6_OH, G_5.5_COOH) on cell viability was assessed on HER2-positive breast cancer cells, SKBR3 and ZR75. In addition, MCF10A cells were used as control. G_6_ cationic PAMAMs (G_6_NH_2_) showed the most significant dose-dependent reduction of cell viability by 5.1% ± 2.14 (*p* < 0.001) and 5.75% ± 0.87 (*p* < 0.01) in SKBR3 and ZR75, respectively at a concentration of 10 µM (Fig. 1A and B). However, treatment with G_4_NH_2_ PAMAMs did not show a significant reduction in cell viability at concentrations below 5 µM (Fig. 1C and D). On the other hand, G_6_OH and G_5.5_COOH PAMAMs were less effective compared to G_6_NH_2_, as they reduced cell viability of SKBR3 down to 26%±7.21 and 38%±4.73, respectively (*p* < 0.001) (Fig. 1E-H). Interestingly, less reduction rate in cell viability was noted in MCF10A cells treated with cationic PAMAMs after 48 h of exposure compared to cancer cells (Fig. 1K and L). Moreover, in comparison with our positive control, lapatinib, cells were more sensitive to G_6_ cationic PAMAMs (Fig. 1I and J).

**Fig. 2:**
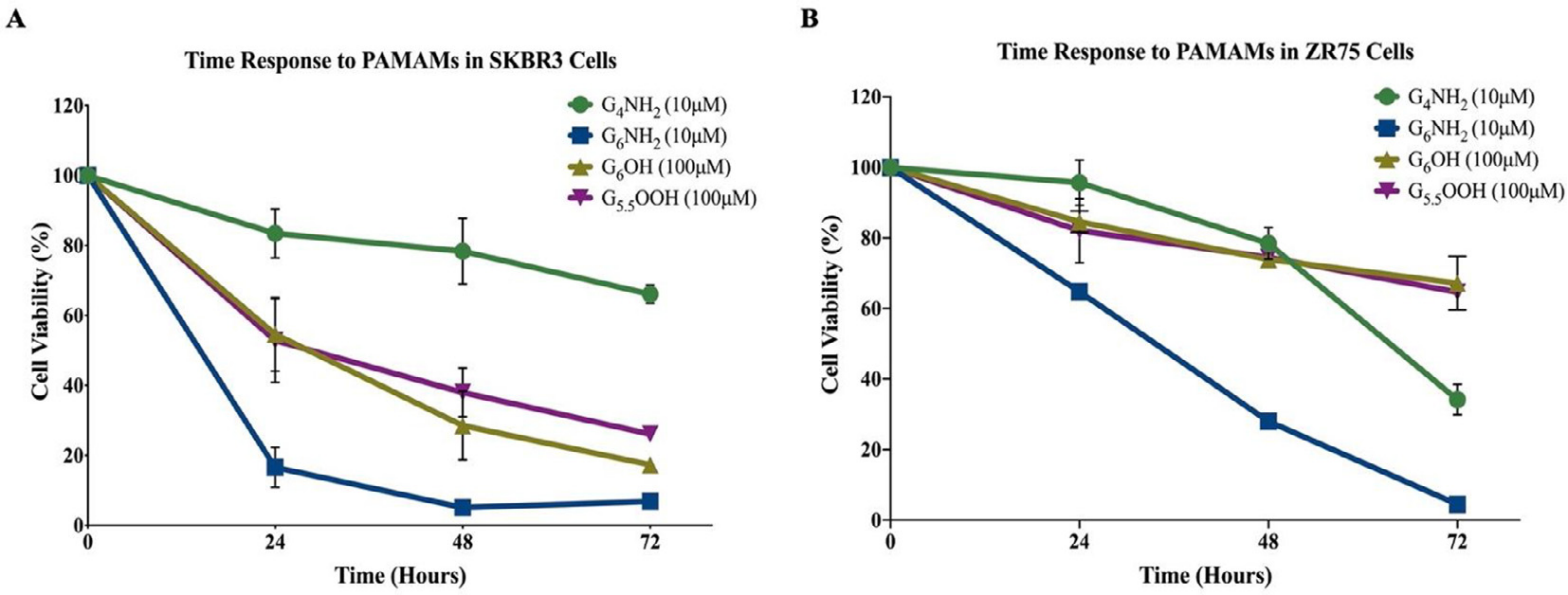
**(A and B).** Time response to treatment with PAMAM dendrimers. Time response to PAMAM dendrimers was investigated in **(A)** SKBR3 and **(B)** ZR75 cells. Cells were treated with G_4_NH_2_ (10 µM), G_6_NH_2_ (10 µM), G_6_OH (100 µM) and G_5.5_COOH (100 µM). Cell viability was assessed after 48 h of treatment. Data are presented as a percentage of treatment relative to the control (Mean ± SEM; n = 3).

The impact of PAMAM dendrimers on cell viability was time dependent as well, since the inhibitory effect of G_6_NH_2_ PAMAMs decreased to 6.87% and 2.64% in SKBR3 and ZR75 cells, respectively after 72 h of exposure (Fig. 2).

**Fig. 3:**
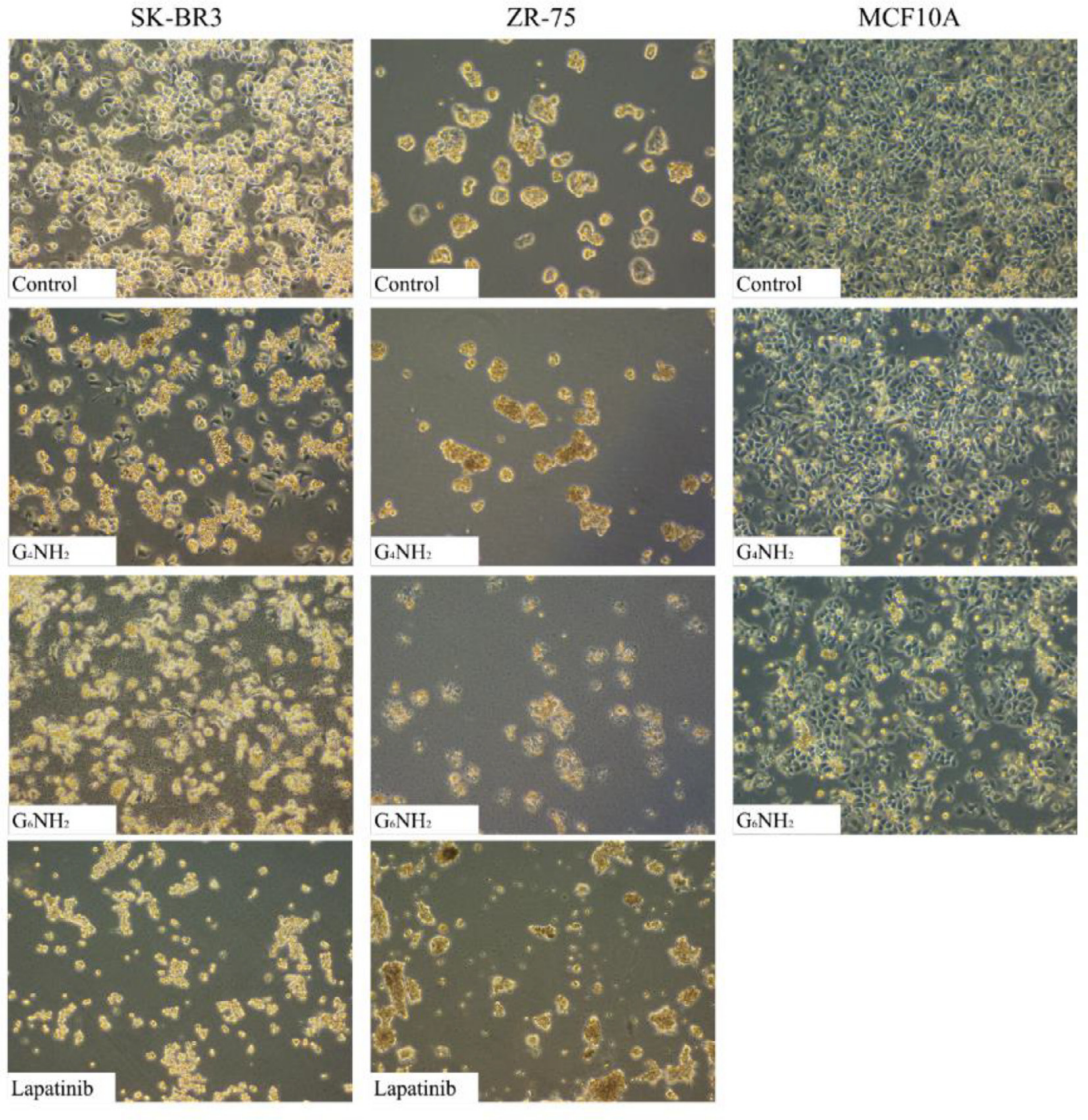
Morphological changes induced by cationic PAMAM dendrimers and lapatinib. SKBR3, ZR75 and MCF10A cells were treated with 5 µM of G_4_NH_2_ and G_6_NH_2_. Images were taken at a magnification scale of 10X following 48 h of treatment (n = 3). SKBR3 and ZR75 cells were treated with lapatinib and morphological images were taken at a magnification scale of 10X following 48 h of treatment (n = 3).

Subsequently, we examined cell morphology of SKBR3 and ZR75 in addition to MCF10A cell lines under the effect of PAMAMs. In the absence of treatment, SKBR3 and ZR75 cells display a round morphology and disorganized multilayered cells. However, following treatment with G_6_ cationic PAMAMs SKBR3 and ZR75 cancer cells lose their shape, cellular membrane integrity, and cell adhesion in comparison with their matched control and MCF10A cells, which showed less morphological changes (Fig. 3). Exposed cells start detaching from the surface of the dishes, indicating cell death in SKBR3, ZR75 and to a lesser extent in MCF10A cells as shown in Fig. 3. Furthermore, we observed that morphological changes triggered by G_6_NH_2_ PAMAMs in cancer cells were more pronounced when compared to treatment with lapatinib (Fig. 3). On the other hand, other types of PAMAMs do not induce a noticeable effect on cell morphology in comparison with control cells (Supplementary Fig. 1).

**Fig. 4:**
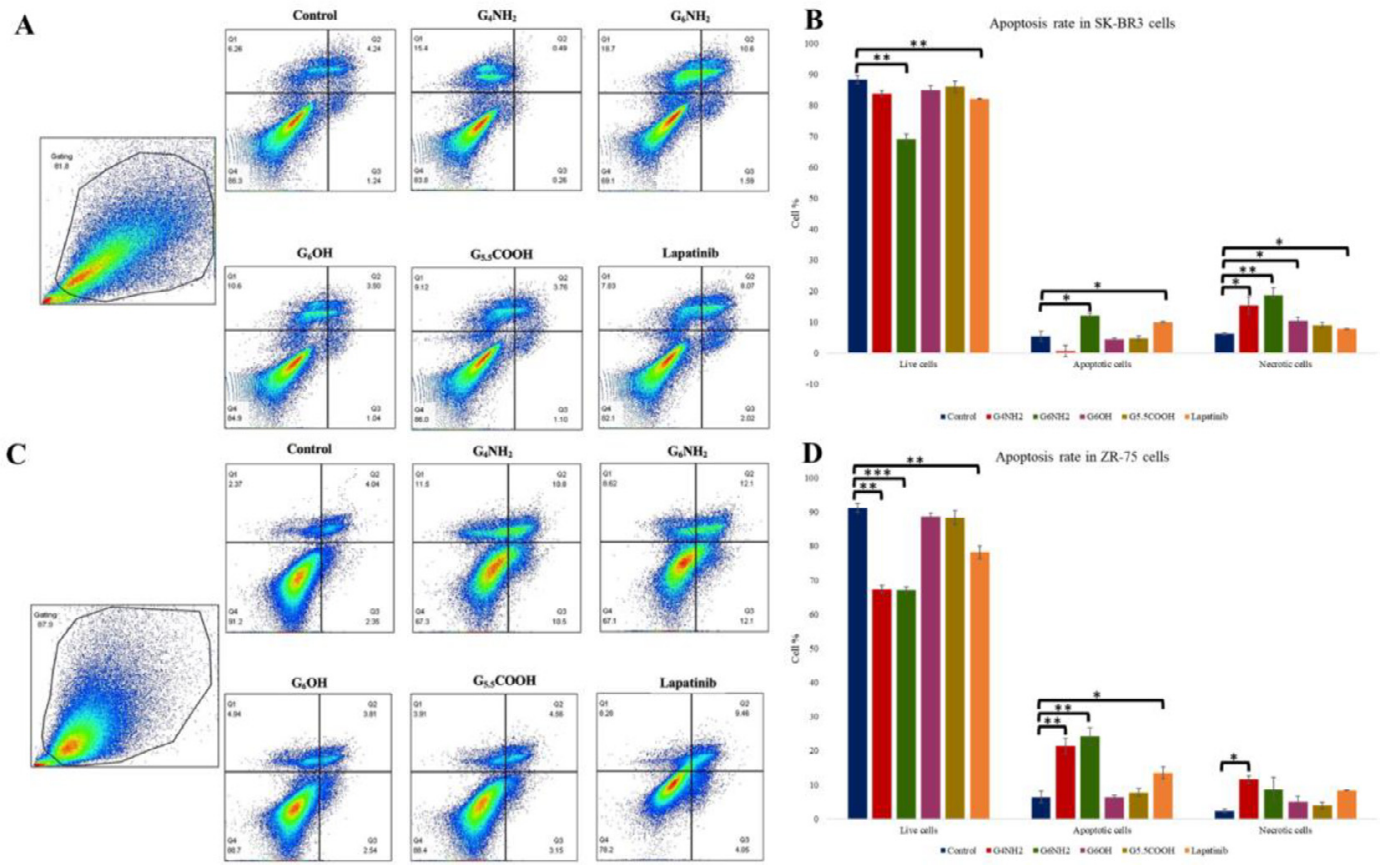
**(A -D).** Induction of apoptosis by PAMAM dendrimers in **(A and B)** SKBR3 and **(C and D)** ZR75 cells as determined by Annexin V apoptosis assay. Cells were treated with 5 µM of G_4_NH_2_, G_6_NH_2_, G_6_OH and G_5.5_COOH PAMAMs. Statistical analysis was performed using one-way analysis of variance (ANOVA). Tukey’s post-hoc test was conducted to compare treatment groups and results were stated as *statistically significant when *p* < 0.05 compared to the control. * p < 0.05, **p < 0.01, and *** p < 0.001.

Next and based on our data of cell viability and morphology, we examined the outcome of PAMAMs on cell apoptosis in HER2-positive cancer cell line, SKBR3 and ZR75, using Annexin V assay. Our analysis showed consistently that cells treated with G_6_ cationic PAMAMs have a significantly higher apoptotic rate in comparison with the positive control, lapatinib (Fig. 4). Also, the rate of apoptosis produced by G_6_ cationic PAMAMs was higher than that seen in their matched control as well as other types of PAMAMs; neutral and anionic (Fig. 4). While G_4_ cationic PAMAMs induced necrotic effects in both cell lines (Fig. 4). However, the patterns produced by other types of PAMAMs did not differ significantly from their control (Fig. 4).

**Fig. 5:**
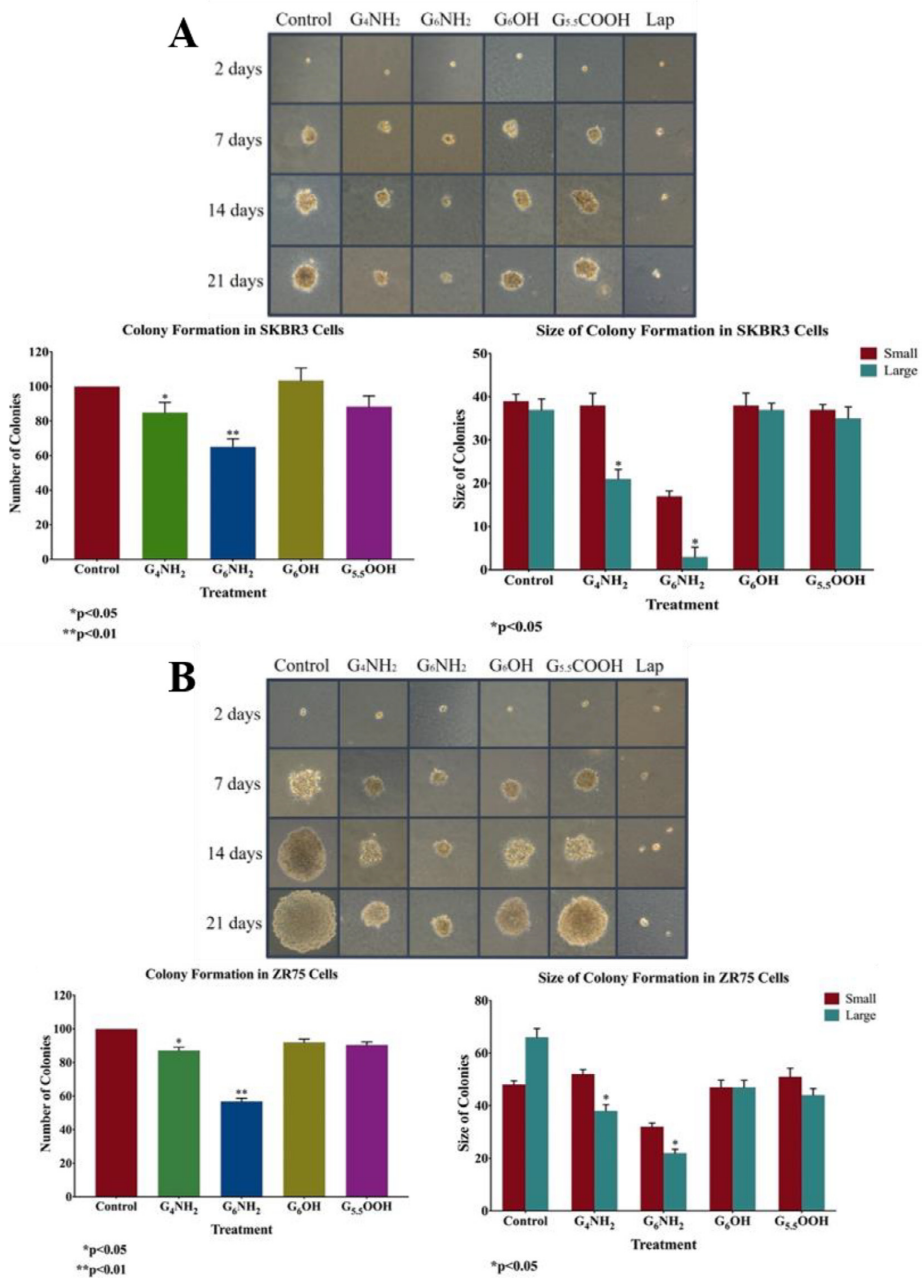
**(A and B).** Effect of PAMAM dendrimers on colony formation, in soft agar, in HER2-positive breast cancer cell lines, **(A)** SKBR3 and **(B)** ZR75. Cells were treated with 5 µM of G_4_NH_2_, G_6_NH_2_, G_6_OH and G_5.5_COOH PAMAMs. PAMAM dendrimers inhibit colony formation of SKBR3 and ZR75 in comparison with their matched control cells. Colonies were counted manually and expressed as a percentage of treatment relative to the control (Mean ± SEM). Statistical analysis was performed using one-way analysis of variance (ANOVA). Tukey’s post-hoc test was conducted to compare treatment groups and results were stated as *statistically significant when *p* < 0.05 compared to the control. * p < 0.05, **p < 0.01, and *** p < 0.001.

Additionally, we examined the colony formation of SKBR3 and ZR75 cells in soft agar, under the effect PAMAMs. Our data showed a significant decrease in the number of colonies for both cell lines treated with G_6_NH_2_ PAMAMs compared with their matched controls, followed by cells treated with G_4_NH_2_ PAMAMs as shown in Fig. 5. In contrast, treatment with G_6_OH and G_5.5_COOH PAMAMs did not affect the size and number of colonies in comparison with their controls (Fig. 5). Quantification analysis revealed a significant decrease in colony number and size in cells treated with cationic polymers (*p* < 0.05) in comparison to treatment with G_6_OH and G_5.5_COOH PAMAMs and their controls (Fig. 5). This indicates that cationic G_6_ PAMAMs significantly suppress colony formation of HER2-positive breast cancer and probably tumor growth *in-vivo.*

**Fig. 6:**
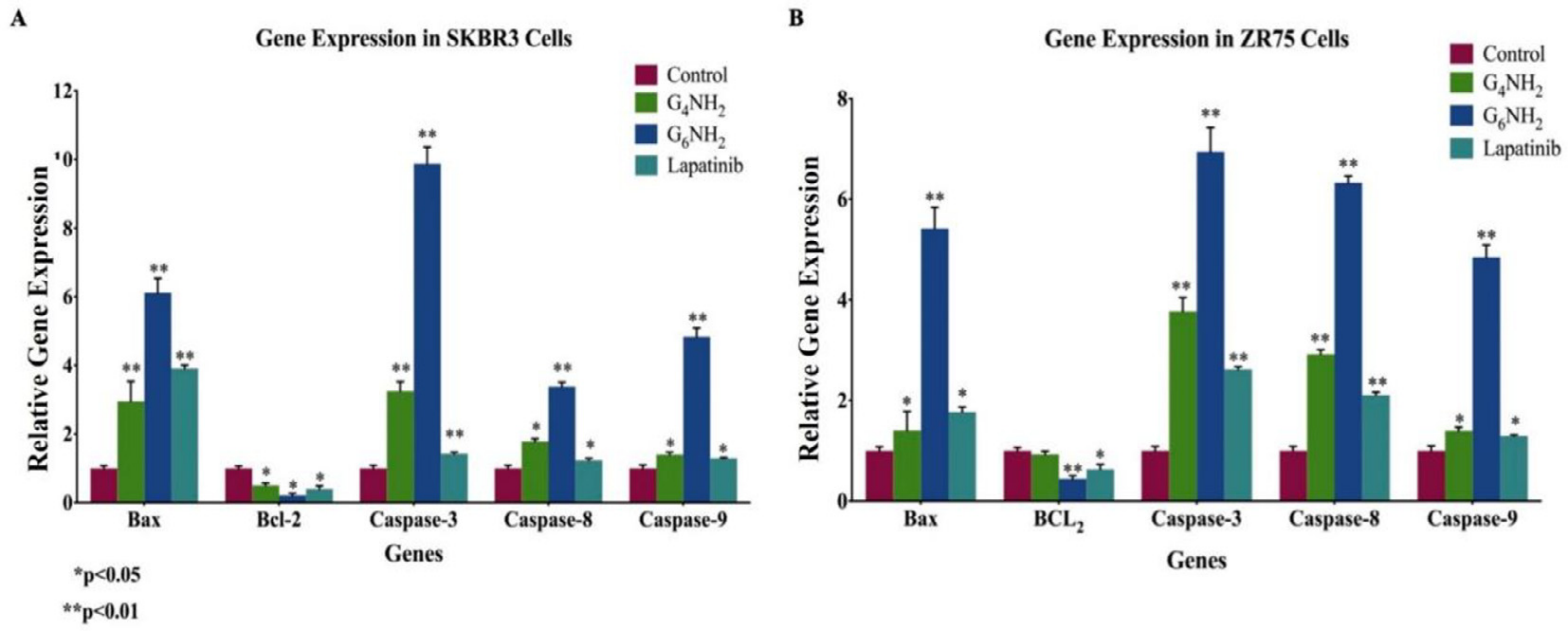
**(A and B).** RNA expression and molecular mechanisms of PAMAM dendrimers inhibitory actions in **(A)** SKBR3 and **(B)** ZR75 cell lines. PAMAMs induce deregulation of pro-apoptotic markers (Bax, Caspases-3, −8 and −9) in comparison with their control and inhibit anti-apoptotic markers (Bcl-2). Cells were treated with: G_4_NH_2_ and G_6_NH_2_ PAMAMs and lapatinib. GAPDH was used as a control for gene expression in this assay. Data are presented as a percentage of treatment relative to the control (Mean ± SEM; n = 3). Statistical analysis was performed using one-way analysis of variance (ANOVA). Tukey’s post-hoc test was conducted to compare treatment groups and results were stated as *statistically significant when *p* < 0.05 compared to the control. * p < 0.05, **p < 0.01, and *** p < 0.001.

**Fig. 7:**
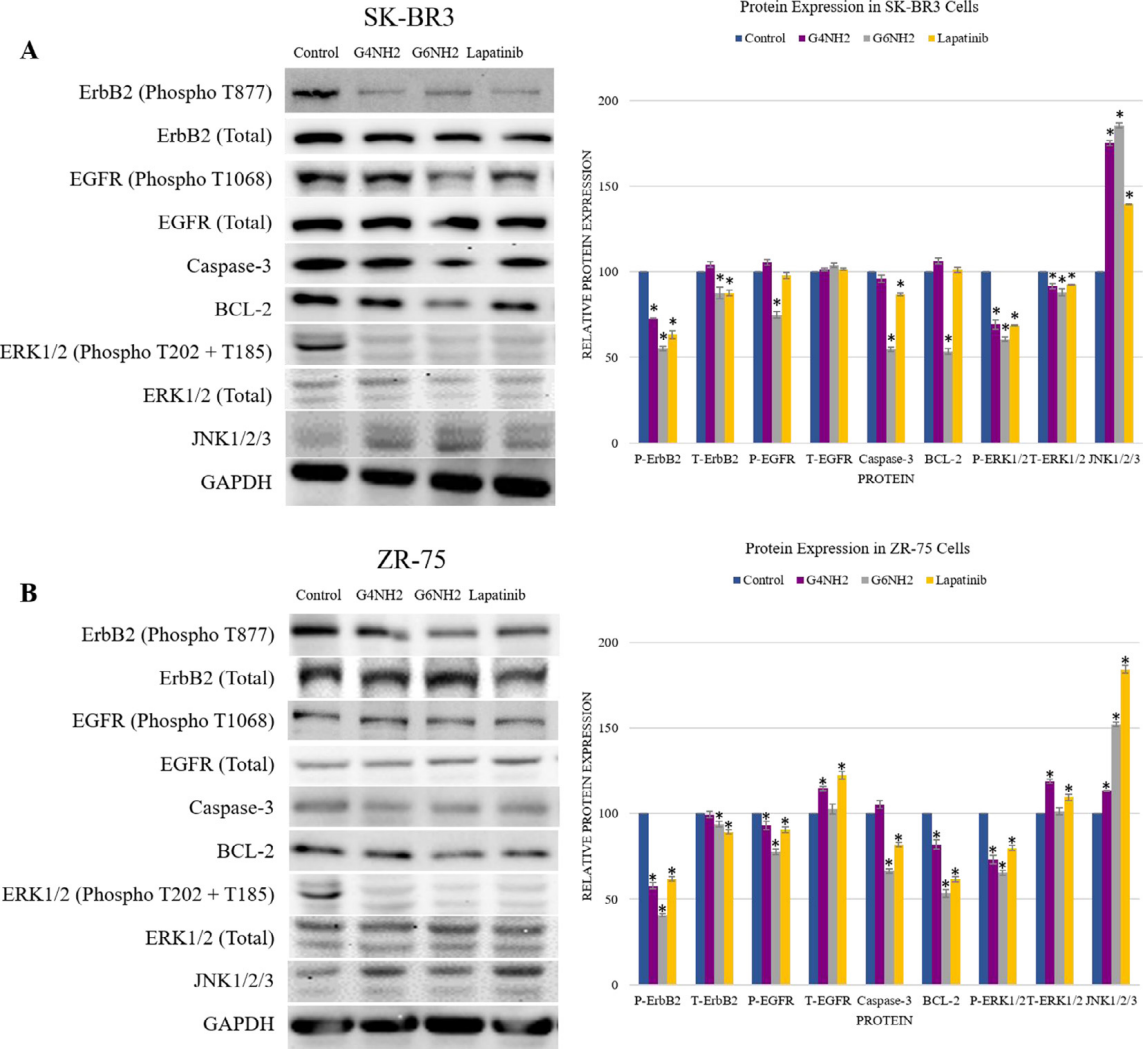
**(A and B).** Protein expression and molecular mechanisms of PAMAM dendrimers inhibitory actions in **(A)** SKBR3 and **(B)** ZR75 cell lines. Cells were treated with: G_4_NH_2_, G_6_NH_2_ PAMAMs, and lapatinib. GAPDH was used as a control for the loaded amount of the protein in this assay. Data are presented as a percentage of treatment relative to the control (Mean ± SEM; n = 3). Statistical analysis was performed using one-way analysis of variance (ANOVA). Tukey’s post-hoc test was conducted to compare treatment groups and results were stated as *statistically significant when *p* < 0.05 compared to the control. * p < 0.05, **p < 0.01, and *** p < 0.001.

Based on the data above, since anionic PAMAMs (G_6_OH and G_5.5_COOH) did not induce cell apoptosis using the Annexin V assay, no further molecular mechanism analysis was performed for these types of PAMAMs. Hence, we studied the expression patterns of key markers of apoptosis in HER2-positive breast cancer cells under the effect of the cationic PAMAMs (G_4_NH_2_ and G_6_NH_2_) and lapatinib; we found enhanced expression of the pro-apoptotic markers (Bax, Caspase-3, 8, and 9) in PAMAM-treated cells compared to their control as shown in Fig. 6, Fig. 7. On the other hand, expression of the anti-apoptotic marker, Bcl-2 was lost when treated with PAMAM dendrimers (Fig. 6, Fig. 7). In detail, it can be seen that in SKBR3 cells, treatment with G_6_NH_2_ upregulates the expression of Bax by 6-folds, while Bcl-2 was reduced by 0.2-fold. Moreover, expressions of Caspases- 3, −8, and −9 were increased by 10-, 3- and 5-folds, respectively. Similarly, for ZR75 cells, treatment with G_6_ cationic PAMAMs upregulates Bax expression by 5.4-folds, while Bcl-2 was reduced by 0.4-fold. Treatment with G_6_NH_2_ also enhanced the expression of Caspases-3 (7-folds), −8 (6-folds), and −9 (5-folds). On the other hand, treatment with lapatinib resulted in a less significant impact on the gene expression compared to G_6_ cationic PAMAM dendrimer (Fig. 6, Fig. 7).

Vis-à-vis the underlying molecular pathways of PAMAMs on cell viability and apoptosis as well as colony formation of HER2-positive breast cancer cells, we assumed that HER1 and 2 activation and c-Jun N-terminal kinase (JNK) in addition to ERK1/2 could have major roles in regulating these events; therefore, the expression patterns of HER1 and 2, as well as JNK1/2/3 and ERK1/2, were explored. Our analysis revealed that PAMAM dendrimers reduce the expression of total and phosphorylated HER1 and 2 compared to their controls in a generation-dependent manner, with the highest effect seen in G_6_ cationic PAMAMs (Fig. 7). More significantly, our data pointed out that PAMAMs induce upregulation of JNK1/2/3 and downregulation of phosphorylated Erk1/Erk2 in both HER2-positive cancer-treated cells in comparison with their control (Fig. 7). In parallel, treatment with lapatinib produced similar effects to PAMAM dendrimers in terms of modulating the protein expression, but they were often less significant compared to G_6_NH_2_ (Fig. 7). Consequently, apoptotic activity in treated breast cancer cell lines was confirmed by analyzing the expression of JNK/ERK pathway.

## 4 Discussion

In this study, we investigated the effect of PAMAM dendrimers on HER2-positive human breast cancer cell lines (SKBR3, ZR75) with regards to cell viability, apoptosis as well as colony formation. Additionally, we analyzed the underlying molecular pathways. The data presented in this study revealed for the first time novel anti-cancer effects of naked PAMAM dendrimers in HER2-positive breast cancer. The experimental screening showed a significant dose and time-dependent growth inhibition in HER2-positive breast cancer cells upon treatment with PAMAM dendrimers, mainly G_6_ cationic PAMAMs, which produced the lowest IC_50_, thus, indicating a role of PAMAM dendrimers as anti-HER2 compounds. Other PAMAMs with neutral or anionic surface chemistry showed a less significant reduction of cell viability compared to cationic PAMAMs suggesting the dependency of this effect on the cationic surface. A similar study by Kuo et al. highlighted the major role of PAMAMs cationic surface in reducing cell viability of cervical cancer cell line; HeLa, as neutralizing the surface charge resulted in a loss of the anti-cancer action [27]. However, a major difference between G_4_ and G_6_ cationic PAMAMs was reported in this study, as the latter was far more effective against HER2-positive cancer cells. This generation-dependency can be due to the highly branched structure of G_6_ PAMAMs as compared to G_4_ PAMAMs and their higher number of terminal amino groups; G_6_ PAMAMs contain 256 surface amino groups [28].

In order to investigate whether the reduction in cell viability induced by PAMAM dendrimers results from inhibition of cell proliferation or due to induced cell death, we assessed the changes in cell morphology. Our data showed that morphological changes induced by cationic PAMAMs in the examined cell lines (SKBR3, ZR75) reflected hallmarks of cell apoptosis; such as membrane loss, as it is well known that PAMAMs can interact with cellular membranes leading to cell lysis [29]. Further morphological examination also showed a high number of cell death in wells treated with G_6_NH_2_ PAMAMs compared to other PAMAMs and their controls, which was confirmed by apoptosis analysis. Several studies focused on the mechanisms of cell death produced by PAMAM dendrimers, particularly apoptosis and necrosis. Our Annexin V apoptosis assay revealed that cationic PAMAMs induced apoptosis (mainly G_6_) and necrosis (mainly G_4_) in SKBR3 and ZR75 cell lines. Similar to our results, it was previously shown that G_4_ cationic PAMAMs induce strong necrotic and weak apoptotic cell death in human acute T-cell leukemia Jurkat cells [30]. Also, G_5_ cationic PAMAMs were found to induce apoptosis and necrosis in both KB cells and RAW 264.7 murine macrophage-like cells [31], [32]. These data emphasize that PAMAMs cationic surface chemistry plays a significant role in inducing apoptotic and necrotic cell death. Other suggested mechanisms for apoptosis induction by cationic PAMAMs in the literature include disruption of mitochondrial membrane potential (MMP) and activation of ATM-mediated DNA damage. Cationic PAMAMs are also capable of triggering apoptosis in oxidative stress-dependent mechanisms, such as increasing the production of intracellular ROS [18], [31], [33], [34].

On the other hand, our RT-PCR and western blot data show an increase of Bax, Caspases −3, −8 and −9 in PAMAM-treated cells compared to the control. In addition, our data pointed out that the expression of anti-apoptotic marker, Bcl-2 was lost when treated with PAMAM dendrimers suggesting that PAMAM dendrimers induce apoptotic activity in breast cancer cell lines. It has been reported that Bcl-2 homodimers inhibit apoptosis; however, Bax homodimers stimulate cell death [35]. Therefore, heterodimerization between Bax and Bcl-2 and Bax:Bcl-2 determine the sensitivity of cells to apoptosis, whereas Caspase-3 acts as a downstream target of Bax/Bcl-2 control and is involved in triggering apoptosis [35]. We herein report that PAMAMs can inhibit growth and induce apoptosis of human HER2-positive breast cancer cells. This effect is associated with Caspase-3 activation and reduced Bcl-2 expression. Furthermore, PAMAMs can plausibly have induced mitochondrial Bax translocation and loss of Bcl-2 expression, thus, indicating Caspase-dependent pathways are involved in PAMAM-induced apoptosis. In addition, the anti-apoptotic marker Bcl-2 is lost in treated cell lines, indicating Bcl-2/Bax/Caspase-3 regulated cell death through JNK inactivation. These findings correspond with our previous report, as we showed that cationic PAMAMs induce apoptosis by increasing the expression of Caspase-3 and Bax as well as inhibiting the anti-apoptotic marker Bcl-2, during early stages of normal development [36].

Furthermore, regarding the molecular pathways of PAMAM dendrimers on our cell line models, we investigated the downstream target of HER1 and 2 stimuli, JNK. For the interaction between the activation of HER1 and 2 receptor and its downstream pathways, including JNK, it is known that upregulated HER1 and 2 expression causes homo- or heterodimerization resulting in the phosphorylation of this receptor which further stimulates downstream signaling pathways involved in apoptosis, chemoresistance, cell proliferation, invasion and migration, [37], [38]. Particularly in HER2-positive breast cancer, it was stated that the downregulation of the JNK pathway drives tumor growth and proliferation [39].

Our study demonstrates that PAMAM dendrimers inhibit expression of the HER1 and 2 receptors while mostly affecting their phosphorylation as well as one of their main downstream targets JNK. We found that cationic PAMAM dendrimers enhance JNK1/2/3 activity in HER2-positive breast cancer cells. Similar to our data, studies have shown loss of JNK1/2/3 to significantly increase tumor formation [40] as well as increases cancer cells’ resistance to several anti-cancer agents [41]. Moreover, loss of HER1 and 2 are associated with reducing cellular proliferation and invasion of HER2-positive human breast cancer [42], [43]; this is in concordance with our data of PAMAM-induced decreased cell proliferation and colony formation. We also noticed a reduction in ERK1/2 phosphorylation in accordance with the findings of another study which showed an inhibition of transactivation of HER2, EGFR (HER1), and ERK1/2 caused by treatment with cationic PAMAMs *in vitro* and *in vivo* [19]. ERK1/2 is a downstream signaling of HER1 and 2 receptors, regulating cell proliferation.

Although minor effect was reported in MCF10A cells, the possible toxicity of cationic PAMAMs implies the need for further safety assessment to balance between the desired effects and PAMAMs toxicity. In fact, the toxic effects of cationic polymers and nanomaterials are common in delivery systems due to the high density of the positive charge, which interacts with the negatively charged cellular membranes [44], [45], [46]. Nevertheless, several methods have been proposed to diminish this toxicity, such as PEGylation which can increase the biocompatibility of PAMAMs [47], [48], [49]. Other methods proposed in the literature can minimize the toxicity of cationic PAMAMs while preserving their surface chemistry, such as choosing the optimum route of administration. For example, the intratracheal and intraperitoneal pathways were found to be safe in avoiding inflammatory responses caused by cationic PAMAMs compared to the intravenous route by slowing their release [20], [50]. Moreover, a targeted delivery system of PAMAMs linked to trastuzumab was also proposed by different studies, which creates a selectivity towards HER2-positive breast cancer cells rather than other cells [21], [51], [52].

Our results show that the effects of cationic G_6_ PAMAMs are comparable with lapatinib, a well-known anti-HER2 drug. Furthermore, our preliminary data regarding the combination of lapatinib and G_6_NH_2_ PAMAMs show that it was more effective than treatment with lapatinib alone. Concordantly, it was previously stated that PAMAM dendrimers enhance tumor inhibitory effects when co-administered with other anti-cancer compounds [27], [53], [54]. However, the impact of our preliminary data regarding the combination of lapatinib and G_6_NH_2_ PAMAMs on HER2-positive breast cancer cells was less than the effect of G_6_NH_2_ PAMAMs alone. This could be due to the partial neutralization of PAMAMs surface charge upon mixing with lapatinib, which can be avoided by applying each compound solely with an appropriate time interval. Combined, our findings suggest that PAMAM dendrimers, particularly cationic types, represent potentially effective compounds for the treatment of HER2-positive breast cancer and should be subjected to subsequent developmental stages.

## 5 Conclusions

This study reports the effect of PAMAMs on HER2-positive breast cancer and its underlying mechanism. Our anti-cancer screening identified PAMAM dendrimers as promising anti-HER2 compounds with the most effect seen in G_6_NH_2_, corresponding with the general trend regarding the extent of PAMAMs activities; surface chemistry and generation-dependency. Furthermore, this study brings about novel therapeutic potential by demonstrating the induced inhibition of HER1 and 2 as well as ERK/JNK activation by PAMAMs in human breast cancer cells. We believe that PAMAM dendrimers might act as candidate therapeutic agents based on their anticancer activity which can pave the way for potential more advanced therapeutic approaches in breast cancer management, especially HER2- positive cases. Future studied are planned to explore the molecular pathway of cancer cells’ death induced by PAMAM dendrimers as well as their intrinsic toxicity on primary cells. Taken together, PAMAM dendrimers may become a new potential class of anti-HER2 agents, either to act solely or to add benefit to current treatments.

## CRediT authorship contribution statement

**Hadeel Kheraldine:** Methodology, Validation, Formal analysis, Data curation, Writing - original draft. **Ishita Gupta:** Methodology, Validation, Data curation, Writing - review & editing. **Hashim Alhussain:** Methodology. **Aayesha Jabeen:** Methodology, Validation. **Farhan S. Cyprian:** Methodology, Validation. **Saghir Akhtar:** Conceptualization, Writing - review & editing. **Ala-Eddin Al Moustafa:** Conceptualization, Writing - review & editing, Funding acquisition. **Ousama Rachid:** Conceptualization, Writing - review & editing, Funding acquisition.

## Declaration of Competing Interest

The authors declare that they have no known competing financial interests or personal relationships that could have appeared to influence the work reported in this paper.

## Figures and Tables

**Supplementary Fig. 1:**